# Novel Cases of Poisoning

**Published:** 1890-10

**Authors:** 


### NOVEL CASES OF POISONING.

Two Cases of Poisoning with Natrum Nitrosum. By Dr. Collischorm.—Two patients received by mistake Natrum nitrosum 5.0 : 150.0 pro dosi instead of Natrum nitricum, a tablespoonful every hour. The first patient was attacked with diarrhœa, fainting, bitter eructations, heavily coated tongue, slight cyanosis, and on the chest an eruption simulating roseola syphilitica; the urine contained traces of albumen; during the night copious, alvine discharges with faintishness. During the next day—he took so far 3.0—intensive cyanosis set in, especially of the mouth, tongue, and fauces; the eruption spread to the thighs and abdomen. The dark-yellow urine contained copiously urates. The following day he took 2.5 grm., the cyanosis diminished, and the measly exanthem spread all over the body, excepting the face. As soon as the drug was left off, cyanosis and all other troubles disappeared. The second patient had the cyanosis; diarrhœa and faintings in an aggravated form, so that collapse threatened. Large quantities of strong coffee and omission of the drug removed the symptoms of poisoning. Both cases clearly showed the symptoms of acute gastro-enteritis and the formation of methæmoglobine; the absence of any renal disturbance was noticed, and it seems probable that the salt is rapidly carried through the kidneys and discharged by vomiting and diarrhœa, hence the rapid disappearance of the dangerous symptoms.—*Centralbl. f. Kl. Med.* 16, '90.

Poisoning by Potatoes.—Surgeon Cortial received during two days over a hundred soldiers into the hospital, and all complained of headache, dilated pupils, visual disturbances, colic, diarrhœa, epigastric pains, thirst, fever, dizziness, nausea, perspiration, spasms. Suspecting poisoning by food, it was soon found that the purveyor delivered instead of new potatoes old ones full of excrescences. Such shoots and green potatoes contain too much solanin. The soldiers were sick from four to eight days. The first symptoms appeared eight to ten hours after the meal, and milk-diet was the only treatment employed. —*Centralbl. f. Ther.* 3, 1890.

Poisoning by Oysters.—At Minragun, in Japan, whose inhabitants live mostly on fishes, lately appeared such an extensive mortality that the government sent a commission there to find out its cause. Shortly before the epidemic appeared the fishermen discovered a new oyster-bed, and every one partook of the feast, raw or cooked. Experiments with these oysters demonstrated that they act as a poison on animals, and chemical analysis proved it to be tyrotoxine. A few years ago a similar fatal epidemic devastated Wilhelmshafen, and it was found that the bed was on a spot where the detritus of the city emptied itself into the river, and that the oysters or mussels could be made edible again by transplanting them into pure and healthy water. *Sublata causa tollitur effectus.*—*A. M. Centr. Zeit.* 32, '90.

Powdered charcoal mixed with brandy, strong black coffee mixed with the juice of half a lemon (a capital prescription also in some malarious intermittents), vinegar and water in equal quantities are highly praised as antidotes. Still I cured cases of poisoning by mussels with Carbo-vegetabilis 30th or 200th potency in water, a dessertspoonful every five or ten minutes.

